# Jack of many trades: Multifaceted role of neuropilins in pancreatic cancer

**DOI:** 10.1002/cam4.1715

**Published:** 2018-09-14

**Authors:** Pratiek N. Matkar, Eric D. Jong, Ramya Ariyagunarajah, Gerald J. Prud'homme, Krishna K. Singh, Howard Leong‐Poi

**Affiliations:** ^1^ Division of Cardiology Keenan Research Centre for Biomedical Science Li Ka Shing Knowledge Institute of St. Michael's Hospital Toronto Ontario Canada; ^2^ Institute of Medical Science University of Toronto Toronto Ontario Canada; ^3^ Department of Biology University of New Brunswick Fredericton New Brunswick Canada; ^4^ Windsor University School of Medicine Cayon Saint Kitts and Nevis; ^5^ Laboratory Medicine Keenan Research Centre for Biomedical Science Li Ka Shing Knowledge Institute of St. Michael's Hospital Toronto Ontario Canada; ^6^ Laboratory Medicine and Pathobiology University of Toronto Toronto Ontario Canada; ^7^ Vascular Surgery Keenan Research Centre for Biomedical Science Li Ka Shing Knowledge Institute of St. Michael's Hospital Toronto Ontario Canada; ^8^ Department of Pharmacology and Toxicology University of Toronto Toronto Ontario Canada; ^9^ Department of Surgery University of Toronto Toronto Ontario Canada; ^10^ Department of Medical Biophysics Schulich School of Medicine and Dentistry University of Western Ontario London Ontario Canada

**Keywords:** Neuropilin, Pancreatic Cancer, Pancreatic Ductal Adenocarcinoma, Semaphorin, Vascular Endothelial Growth Factor

## Abstract

Neuropilins (NRPs) have been described as receptors for class 3 semaphorins and coreceptors for a plethora of ligands, such as members of the vascular endothelial growth factor (VEGF) family of angiogenic cytokines and transforming growth factor (TGF). Initial studies using genetic models have indicated that neuropilin‐1 (NRP‐1) is essential for axonal guidance during neuronal and cardiovascular development, regulated via semaphorins and VEGF, respectively, whereas the other homolog of neuropilin, NRP‐2, has been shown to play a more specific role in neuronal patterning and lymphangiogenesis. Pancreatic ductal adenocarcinoma (PDAC) remains a significant cause of cancer mortality with the lowest five‐year survival rate compared to other types of cancer. Recent findings have indicated that NRPs are abundantly expressed in pancreatic cancer cell lines and pancreatic tumor tissues, where they mediate several essential cancer‐initiating and cancer‐promoting functional responses through their unique ability to bind multiple ligands. Specifically, NRPs have been implicated in numerous biological processes such as cancer cell proliferation, survival, invasion, and tumor growth. More recently, several other protumorigenic roles mediated by NRPs have emerged, advocating NRPs as ideal therapeutic targets against PDAC.

## 1 INTRODUCTION

**Figure 1:**
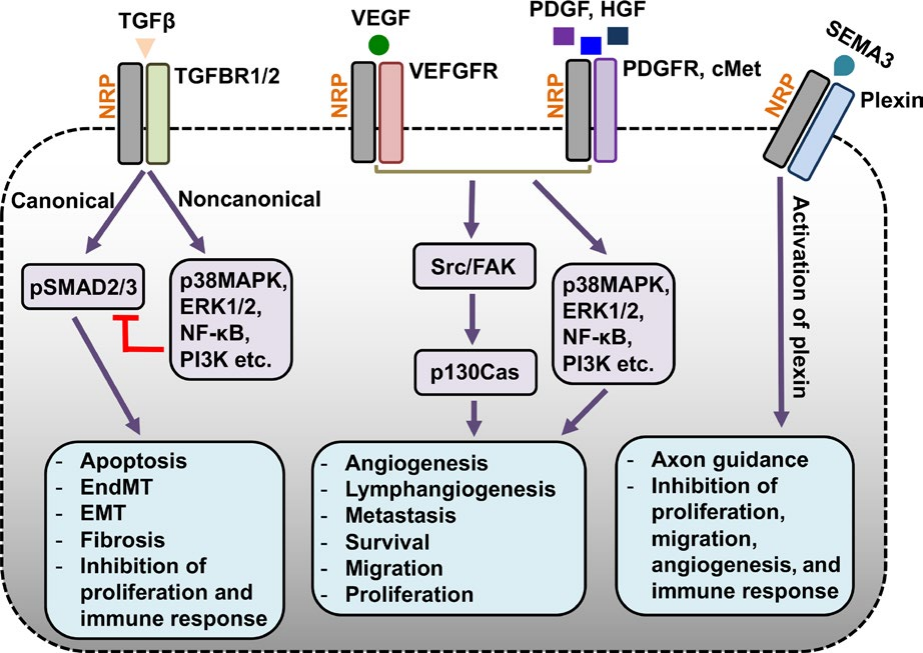
Interactions and functions of NRPs. NRPs interact with five major types of soluble ligands such as TGF‐β1, VEGF, hepatocyte growth factor (HGF), platelet‐derived growth factor (PDGF)‐BB, and semaphorin 3 (SEMA3) family and their corresponding signaling receptors as a receptor or coreceptor. The functions mediated by growth factor (GF) pathways (HGF, PDGF, etc.) overlap with several other cellular functions. The TGF‐β canonical signaling pathway mainly yields antiproliferative, cell transformative, and immunosuppressive effects, which can be inhibited through TGF‐β noncanonical signaling.^16^, ^65^ NRPs activate p130Cas adaptor molecule upon binding to GFs and can inhibit canonical SMAD2/3 signaling, while preferring noncanonical signaling. Additionally, SEMA3 ligands crosstalk with NRPs and plexins in order to regulate axonal guidance and suppress other cellular functions such as proliferation, migration, and angiogenesis^16^

Neuropilins (NRPs) are 130‐140 kDa single‐pass transmembrane glycoproteins that play critical roles during embryonic development, cell immunity, and cancer.^1^, ^2^, ^3^ They are nontyrosine kinase receptors that exhibit multifunctionality and are expressed not only by cancer cells, but also other normal cell types.^4^, ^5^, ^6^ Neuropilin‐1 (NRP‐1) and neuropilin‐2 (NRP‐2) are the two highly conserved homologs in vertebrates that also exist as NRP‐1/NRP‐2 heterodimers.^7^ They were initially studied in neurons as a receptor for class 3 semaphorins (SEMA3) that interact with plexins to facilitate axonal guidance during neuronal development (Figure 1).^8^ The pleiotropic nature of the NRPs has been demonstrated through their involvement in other signaling pathways such as angiogenesis and lymphangiogenesis.^9^ A notable interaction includes enhanced signaling transduction of VEGF‐A_165_ on VEGFR2 that increases the extent of angiogenesis (Figure 1). On the other hand, NRP‐2, not NRP‐1, is a coreceptor for VEGFR3, which is primarily involved in lymphatic endothelial cell function.^10^ Further, NRPs enhance the effect of various other growth factors and signals due to underlying physiological or pathological conditions. NRPs also mediate the interaction between active transforming growth factor‐beta 1 (TGF‐β1), its receptors (TGFBR1 and TGFBR2), and coreceptor betaglycan (TGFBR3) (Figure 1). Other growth factors, such as FGF (fibroblast growth factor), EGF (epidermal growth factor), and HGF (hepatocyte growth factor), share an affinity to the NRPs as well.^5^, ^11^, ^12^, ^13^ However, only formal binding assays between NRPs and certain members of the SEMA and VEGF families have been performed.^14^, ^15^ In addition, it has been well documented that although some ligands interact with both NRPs, others are specific to each homolog.^16^ Finally, the precise signaling function mediated by NRPs, independent of other signaling receptors, remains unclear despite their ability to bind a variety of ligands and recruit adaptor proteins (Figure 1).

**Figure 2:**
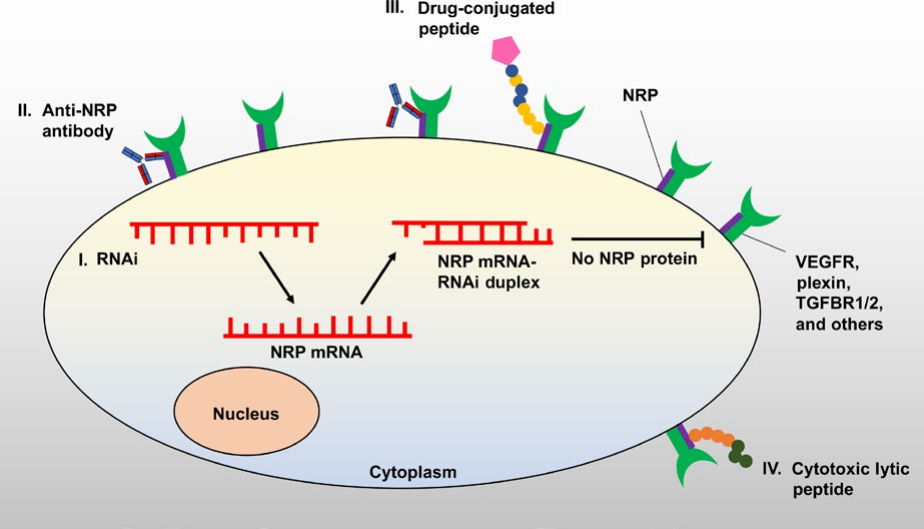
Strategies for anti‐NRP cancer therapy. (I) RNA interference is a biological process in which small RNA molecules (siRNAs, shRNAs, or microRNAs) inhibit gene expression or translation, by binding to the targeted mRNA molecules. Accordingly, synthetic RNAi molecules or vector‐based RNAi directed against NRPs can be introduced into cancer cells to inhibit NRP expression and function. (II) The NRP‐1 human monoclonal antibody has been tested in vitro, in vivo*,* and human clinical trials. Binding of the antibody will impede binding of other ligands to NRPs and thus block the subsequent signaling pathways. (III) Peptides with a C‐terminal consensus R/KXXR/K motif (K‐Lysine, R‐Arginine), preferentially with a C‐terminal arginine (R) or occasionally lysine (K), bind to the b1 domain of NRP‐1. Accordingly, administration of drug‐loaded peptides could result in enhanced penetration of a range of drugs into the cancer cells. (IV) Hybrid lytic peptides containing NRP‐binding sequence conjugated with lytic‐type peptides could be introduced into the cancer cells to induce a cytotoxic effect

Recent findings have indicated that NRPs may play significant biological roles in disease‐related processes such as cancer. Pancreatic ductal adenocarcinoma (PDAC), commonly referred to as pancreatic cancer, remains one of the leading causes of cancer mortality with the lowest five‐year survival rate compared to other cancer types.^17^, ^18^, ^19^ A notable feature of PDAC is the tumor stroma/desmoplasia, which is described as tumor fibrosis surrounding epithelial cells that display variable ductal differentiation.^20^, ^21^, ^22^, ^23^ In effect, cancer‐associated fibroblasts (CAFs) and other stromal cells account for the majority of the PDAC tumor.^24^, ^25^ As shown by the recent evidence, CAFs are capable of altering the tumor microenvironment by releasing oncogenic and angiogenic factors such as TGF‐β.^24^, ^26^, ^27^ Extensive tumor desmoplasia in PDAC can significantly impair drug delivery due to the extracellular matrix and high interstitial pressure within the tumors.^28^, ^29^, ^30^, ^31^ NRPs have been implicated in numerous biological processes such as cell proliferation, survival, invasion, and pancreatic tumor growth. More recently, additional protumorigenic roles mediated by NRPs have emerged, advocating that NRPs could be ideal therapeutic targets against pancreatic cancer. In this review, we will focus mainly on the important protumorigenic functions mediated by the NRPs in pancreatic cancer. We will also investigate the potential of NRPs as therapeutic targets and discuss some of the strategies for anti‐NRP cancer therapy (Figure 2).

## 2 NEUROPILINS IN PANCREATIC CANCER

In the normal pancreas, NRP‐1 is absent and NRP‐2 is only detected in the endocrine islets and in some acinar cells; however, both NRP‐1 and NRP‐2 are highly expressed in pancreatic cancer.^32^ Despite numerous studies exhibiting the association of NRP overexpression with the tumorigenic properties of PDAC, Grey and group described a differential role of NRP‐1 whereby its downregulation promoted tumor growth.^33^ Nonetheless, it has been demonstrated that tumor angiogenesis, advanced tumor‐node‐metastasis stage, pT stage, node invasion, and dismal postoperative survival are associated with increased NRP‐1 expression in PDACs.^34^ While PDAC also overexpresses NRP‐2, it has not been studied as much as NRP‐1.

In addition to NRP‐1, SEMA3a and plexins are overexpressed in pancreatic cancer and are correlated with poor patient outcome. It is suggested that multiple pathways involving Ras‐related C3 botulinum toxin substrate 1, glycogen synthase kinase 3 beta, and p42/p44 mitogen‐activated protein kinases (MAPK) are responsible for the invasiveness of pancreatic cancer cells upon SEMA3a stimulation.^35^ However, further investigation shows that this process is independent of E‐cadherin to N‐cadherin switch, MMP‐9, and VEGF induction.^35^ Interestingly, the influence of NRP‐1 on tumorigenesis is dependent on the genetic status of *K‐Ras*.^36^ More recently, it has been discovered that transmembrane glycoprotein Mucin 1 is associated with NRP‐1 and is responsible for inducing VEGF signaling as well as angiogenesis in the tumor environment.^37^ The following sections of the review will shed light upon the important functions played by NRPs that contribute to some of the cancer hallmarks as described by Hanahan and Weinberg as well as other cancer‐promoting events.^38^

### 2.1 Functions of NRP‐1 in pancreatic cancer

#### 2.1.1 Proliferation

A deregulated proliferation program is an important acquired characteristic of cancer cells. Li et al^39^ demonstrated enhanced proliferation mediated by endogenously overexpressed NRP‐1 in PANC‐1 pancreatic cancer cell line. Treatment of PANC‐1 with exogenous VEGF increased the extent of proliferation as compared to the untreated control and those treated with an anti‐NRP‐1 antibody. These findings indicated that NRP‐1 mediates cancer cell growth independent of VEGFR. However, it is difficult to draw such a strong conclusion as the expression of functional VEGFRs was only observed in PANC‐1 cells.^40^ On the other hand, overexpression of NRP‐1 in human pancreatic cancer cell line FG, that endogenously expresses NRP‐1 at low levels, resulted in increased proliferation due to constitutive MAPK signaling through extracellular signal‐regulated kinase 1/2 (ERK1/2) and c‐Jun‐NH2‐kinase (JNK).^41^ This suggested that NRP‐1 overexpression induces MAPK signaling, partly by autocrine function, as the group demonstrated increased ERK1/2 and JNK phosphorylation of mock‐transfected cells that were cultured in conditioned medium from NRP‐1‐transfected cells. Although the cause of increased MAPK signaling was not fully elucidated, it is possibly independent of VEGF or SEMA3a.^41^ Further research into identifying other factors mediating this pathway is suggested, as it can provide insight on novel approaches toward developing a therapy, especially as the MAPK pathway promotes cell survival. While many of these in vitro studies demonstrate the particular roles of NRP‐1 in mediating PDAC proliferation, further studies in vivo should be followed to support these observations. Overall, future therapies against PDAC that specifically target NRP‐1 show promise in reducing cell proliferation.

#### 2.1.2 Evasion of apoptosis and chemoresistance

Chemoresistance is a multifactorial phenomenon that contributes to the failure of chemotherapies and dismal survival rates in patients with pancreatic cancer.^42^ Wey et al^41^ demonstrated the role of NRP‐1 overexpression in mediating chemotherapy resistance in PDAC. In their study, cell line FG overexpressing NRP‐1 was less susceptible to anoikis, with cell survival increasing by at least 30%, after gemcitabine or 5‐fluorouracil (5‐FU) treatment. On the other hand, knockdown of NRP‐1 in cell line FG significantly increased susceptibility to gemcitabine and 5‐FU.^42^, ^43^ In another study, NRP‐1 overexpression augmented MCL‐1 expression, indicating a mechanism to evade apoptosis.^41^ Interestingly, constitutive MAPK signaling promoted survival and inhibition of anoikis in other cancer cell types^44^—perhaps, this system may explain the increased survival. Moreover, there are serious implications of NRP‐1‐mediated anoikis resistance including its contribution to the metastatic potential of pancreatic cancer.^45^ Approximately 50% of patients with PDAC that are diagnosed with a nonresectable form are presented with distant metastasis; metastatic recurrence occurs in 70% of patients that undergo radical surgical resection.^46^ As surgical resection seems to be the most promising therapeutic strategy at this moment, a combined approach in the form of an adjuvant anti‐NRP‐1 therapy could shrink the primary tumor making it amenable to surgery.^46^ Although the exact mechanism by which NRP‐1 confers chemoresistance has not been fully elucidated, these key studies indicate that there may perhaps be an association between these two factors. As such, it seems plausible that therapies that target NRP‐1 to control various cancer‐related events might even be more effective due to its additional ability to overcome chemoresistance.

#### 2.1.3 Invasion and migration

Invasion is an important characteristic of cancer that is described by the movement of cancer cells throughout the surrounding tissue and vasculature.^47^ Expression of HGF is typically limited to mesenchymal cells and is secreted as a signal to neighboring epithelial cells.^48^ However, HGF is overexpressed in PDAC, and NRP‐1 modulated HGF‐mediated invasion in NRP‐1‐overexpressing COLO‐357 and PANC‐1 pancreatic cancer cell lines.^49^ It is possible that the invasion is dependent on the c‐Met pathway, as cell invasion was abolished upon c‐Met knockdown.^50^ More interestingly, depletion of NRP‐1 in PANC‐1 cells not only reduced invasiveness, but also their adhesive capabilities. Further, the increased cell adhesion and invasiveness could be attributed to NRP‐1 interaction with integrin β‐1.^51^ However, increased focal adhesion kinase (FAK) phosphorylation was observed in cells expressing low levels of NRP‐1, which warrants caution as it can lead to activated Src, MAPK, and PI3K signaling pathways.^52^ L1 cell adhesion molecule (L1CAM) is a transmembrane cell adhesion molecule associated with NRP‐1 that is expressed by pancreatic cancer cells, as well as tumor‐derived pancreatic microvascular endothelial cells (TuPAMEC).^53^, ^54^ TuPAMEC migration was inhibited upon treatment with an anti‐NRP‐1 antibody, but the effect was stronger when cotreated anti‐L1CAM.^55^ Further, elevated expression of L1CAM in TuPAMEC promoted PANC‐1 cell adhesion to the endothelial cell monolayers as compared to human umbilical vein endothelial cells (HUVECs), implicating that NRP‐1 depletion inhibited tumor metastasis and angiogenesis.^55^ Overall, various studies have demonstrated the role of NRP‐1 in mediating invasion and migration, which can contribute to PDAC metastasis. In any case, when developing therapies that target NRP‐1 to control these events, among others, potential side effects such as increased cell survival via FAK phosphorylation should be considered.

#### 2.1.4 Tumor angiogenesis

Tumor angiogenesis is an essential aspect of tumor growth. Adequate blood supply is important to the tumor to prevent hypoxia or nutrient deprivation. In this event, the tumor exploits its microenvironment by releasing signaling molecules, such as cytokines and growth factors, to activate surrounding quiescent cells, leading to an uncontrollable series of events.^56^ Ben et al^34^ suggested that NRP‐1 upregulation in PDAC was associated with increased angiogenesis due to a significantly positive correlation between NRP‐1 expression and microvessel density in PDAC tissues resected from patients. This may be attributed to enhanced VEGFR2 activation caused by the ability of NRP‐1 to act as a bridge between VEGF and VEGFR. In support of this, Miao et al^57^ demonstrated that NRP‐1 overexpression in rat prostate carcinoma cells resulted in larger tumors and significantly increased microvessel density. However, the exact mechanisms of NRP‐1‐mediated tumor angiogenesis are difficult to pinpoint because NRP‐1 interacts with numerous cancer‐promoting ligands and receptors. To further complicate, NRP‐1 is also expressed by various stromal cells fibroblasts, endothelial cells, and immune cells that can interact with the tumor cells and influence angiogenesis. Although blocking NRP‐1 seemed to enhance the effect of anti‐VEGF therapy in reducing microvessel density of the pancreatic tumor environment,^58^ additional studies are required to investigate whether VEGF signaling alone or in combination with other signaling molecules overexpressed by pancreatic cancer that could interact with NRP‐1 is responsible for enhanced tumor angiogenesis. Despite these findings, antiangiogenic therapies in preclinical models have proved ambiguous in treating less‐vascularized PDAC tumors and may perhaps be more effective as combination therapies.

#### 2.1.5 Energy metabolism

The chronic and aberrant cell proliferation that characterizes cancer cells is attributed not only to the deregulated cell proliferation, but also to the alterations in energy metabolism to facilitate cellular growth and division. Otto Warburg described a peculiar characteristic of cancer cell energy metabolism. Even in the presence of oxygen, cancer cells can reprogram their glucose metabolism by restricting metabolism mainly to aerobic glycolysis.^38^, ^59^ According to the Warburg effect, glycolysis is the main source of energy in cancer cells due to the lack of oxygen in the tumor environment, but glycolysis also occurs even when oxygen is in excess.^60^ As such, it has been demonstrated that NRP‐1 is a positive regulator of VEGF‐induced glycolysis via upregulation of hypoxia‐inducible factor 1α in pancreatic cancer.^61^ Moreover, downregulation of NRP‐1 decreased glycolysis in pancreatic cancer cells. Ultimately, along with our knowledge of differential metabolism, this study suggests the potential of anti‐NRP‐1 therapy to limit tumor progression through modulation of metabolism.

#### 2.1.6 Endothelial‐mesenchymal transition (EndMT)

EndMT is a specific form of epithelial‐mesenchymal transition (EMT) that occurs in endothelial cells. Similar to EMT, EndMT generates activated fibroblasts that contribute to the pathogenesis of PDAC.^62^, ^63^ Genetic analyses of CAFs in the tumor microenvironment show that up to 40% of CAFs were generated via EndMT.^64^ Endothelial cells have the ability to acquire a mesenchymal cell phenotype, characterized by the presence of cell surface markers N‐cadherin, αSMA (alpha smooth muscle actin), and types I/III collagen; concurrently, the loss of endothelial cell surface markers, such as VE‐cadherin and CD31.^62^, ^63^ Matkar et al^65^ highlighted for the first time that TGF‐β1‐induced EndMT in HUVECs is mediated by NRP‐1. Specifically, NRP‐1 knockdown and overexpression reduced and exacerbated EndMT, respectively. Analyses in human PDAC xenografts showed that NRP‐1 expression positively correlated with fibrosis and EndMT in PDAC. This study underlines the potential of novel anti‐NRP‐1 therapy that could reduce pancreatic tumor fibrosis and subsequent progression. If successful, EndMT inhibition may lead to improved drug delivery or delayed PDAC tumor progression due to restricted CAF recruitment. However, the effects of treatment on survival in vivo and the elucidation of a complete mechanistic signaling pathway remain unknown and warrant further investigation.^65^

### 2.2 Targeting NRP‐1 in pancreatic cancer

Epidermal growth factor receptors (EGFR) are overexpressed in pancreatic cancers; however, PDAC is resistant to anti‐EGFR monotherapies and combination therapies.^66^, ^67^ Abolishment of EGFR transduction is compensated by integrin β‐1‐driven Src‐Akt signaling that promotes cell survival. As cell surface integrin β‐1 interacts with NRP‐1, Kim and colleagues developed an EGFR and NRP‐1 dual antibody and have successfully demonstrated inhibition of integrin β‐1 bypass signaling in cetuximab‐resistant PDAC cell lines and in PDAC xenografts. This bypass signaling is independent of *K‐Ras* mutations, making it a promising therapeutic against pancreatic cancers with or without the mutation.^68^

MicroRNAs (miRNAs), nonprotein coding RNAs that are regulators of gene expression, also show promise as tumor biomarkers and therapeutic agents.^69^, ^70^ miR‐1247 is found at low levels in PDAC, positively correlating with a higher recurrence‐free survival of PDAC patients and negatively correlating with tumor grade. Moreover, both NRP‐1 and NRP‐2 are targets of miR‐1247; overexpression of miR‐1247 via treatment with all‐trans retinoic acid resulted in the downregulation of the NRPs. In effect, PDAC cell proliferation was hindered due to G0/G1 cell cycle arrest.^69^ Although miR‐1247 shows promise as a therapeutic target, we need to consider potential side effects, such as the regulation of genes other than the NRPs and potential adverse effects due to redifferentiation of PDAC cells. Similarly, miR‐124‐3p was identified to target the 5′ untranslated region of the NRP‐1 transcript in a glioblastoma multiforme model.^71^ Overexpression of miR‐124‐3p suppressed expression of NRP‐1 which thereby inhibited cell proliferation, migration, and tumor angiogenesis. As there is a wide variety of non‐coding RNAs that regulate gene expression, others that are differentially expressed in PDAC could be identified in the transcriptome; and those such as miR‐1247 may be used as therapeutic molecules.

Other agents such as SEMA3a conjugated with a lytic peptide have been proposed as therapeutic agents against PDAC.^72^ While these hybrid lytic peptides demonstrated cytotoxic effects against NRP‐1‐positive pancreatic cancer cells, they did not affect the normal NRP‐1‐positive cell types. Effects of this therapy in vivo seem promising due to its high specificity for the coreceptor. Additionally, exogenous expression of cyclophilin A reduces NRP‐1 and VEGF mRNA expression levels, which could provide benefits as combination therapy.^73^ However, further research into the mechanism of lytic peptides against cancer cells and the potential side effects is warranted.

Overall, there are various methods by which NRP‐1 can be targeted to treat PDAC, including antibodies, small molecule inhibitors, and synthetic peptides among others. While many of these therapeutic molecules act to reduce the expression, or inhibit the function of NRP‐1 in recipient cells, their mechanisms of action, efficacy, and potential side effects vary. Therefore, one must also consider impeding factors, such as tumor desmoplasia,^28^, ^29^, ^30^, ^31^ when developing therapies.

### 2.3 Functions of NRP‐2 in Pancreatic Cancer

Although not as intensely studied, NRP‐2 is involved in cell survival signaling, migration, invasion, and anchorage‐independent growth in pancreatic cancer cells.^74^ A study exhibiting NRP‐2 knockdown demonstrated significant reductions in these functions in vitro and in vivo using BxPC‐3 cells that naturally overexpresses NRP‐2.^74^ Despite the fact that proliferation was unchanged following NRP‐2 knockdown in BxPC‐3, subcutaneous tumor proliferation in mice was remarkably reduced in the same study. Moreover, it may also be worth investigating the role of the Akt pathway, as activated Akt was reduced in NRP‐2‐depleted cells.^74^ However, the knockdown of NRP‐2 via shRNA did not reduce tumor mass in a murine xenograft model;^75^ differences could be explained by *K‐Ras* mutation in ASPC‐1 compared to wild‐type *K‐Ras* in BxPC‐3. The role of NRP‐2 in mediating the pathogenesis of PDAC has been highlighted in the following sections of this review; however, a greater understanding and knowledge base of NRP‐2 is encouraged before therapies are developed.

#### 2.3.1 Metastasis

In a zebrafish extravasation model using pancreatic cancer cell line ASPC‐1 that expresses NRP‐2, a reduction in NRP‐2 diminished extravasation, which speaks to the metastatic potential conferred by NRP‐2.^75^ In addition, the study suggested that vascular adhesion is mediated by the interaction between NRP‐2 on PDAC cells and α5 Integrin on endothelial cells, as demonstrated by atomic force microscopy. Aside from its function as a coreceptor, investigations into the role of NRP‐2 as an adhesion molecule is encouraged and may lead to different approaches when using NRP‐2 as a therapeutic target.^75^ Interestingly, the depletion of NRP‐2 resulted in the sequestration of biologically active EGFR in endocytic vesicles, which, as a consequence, led to abnormal ERK activation and cell death.^76^ This should be noted because the process is typically associated with cancer drug resistance^77^ and enhanced metastatic potential.^78^ Another study described the role of the VEGF‐C‐NRP‐2 axis in autophagy regulation that confers resistance to drug therapies; downregulation of VEGF‐C and NRP‐2 led to increased drug susceptibility due to an upregulation of autophagy and vesicular trafficking genes, LAMP‐2 and WDFY‐1.^77^ Administration of (‐)‐epigallocatechin‐3‐gallate (EGCG) significantly reduced PI3K/Akt and ERK signaling, as well as pancreatic cancer orthotopic tumor growth, angiogenesis, and metastasis.^79^ Reduction in VEGF and a concurrent increase in SEMA3f expression with decreasing NRP‐2 expression indicated that the NRP‐2 is possibly associated with the antiangiogenic effects of EGCG.^79^ Ultimately, metastasis is a complex program comprised of multiple stages—some of which are mediated by NRP‐2. Thus, it would also be advantageous to be aware of the specific processes that would be affected by NRP‐2‐targeted therapeutics.

#### 2.3.2 Tumor lymphangiogenesis

NRP‐2 also mediates tumor lymphangiogenesis, a significant event that can promote metastasis in the lymphatics via loose and leaky structure of overlapping endothelial cell junctions.^47^ Specifically, tumor cells can escape through the lymphatic capillaries via button junctions and contribute toward metastasis. Outside of the tumor environment, NRP‐2 is normally expressed in lymphatic vessels during embryogenesis. Early metastatic events are caused by the activation of lymphangiogenic coreceptor VEGFR3 when activated by its appropriate ligands VEGF‐C and VEGF‐D.^10^, ^80^ On the other hand, its role in tumor angiogenesis has been described in mouse models, whereby NRP‐2 antibody reduced metastasis in a lung tumor model.^81^ As such, it is recommended that NRP‐2‐mediated lymphangiogenesis in PDAC is investigated to obtain a greater understanding of its metastatic potential via manipulation of the lymphatics.^82^, ^83^

## 3 CONCLUSIONS AND FUTURE DIRECTIONS

Neuropilins are multifunctional membrane proteins that can enhance or modify the signaling pathways through their interactions with various receptors and ligands. Endothelial cells and many cancer cell types express NRPs, including pancreatic cancer wherein most cases they are overexpressed. Moreover, pancreatic tumor progression and/or patient prognosis is correlated to the presence of one or both NRPs. They are implicated in mediating critical functions such as proliferation, survival, tissue invasion and metastasis, angiogenesis, energy metabolism, and cellular transformation events such as EndMT in pancreatic cancer.

Neuropilins are involved in many processes and mechanisms—many of which have not been fully elucidated. Although their interactions with SEMA3 and the VEGF ligands have been well characterized, studies involving HGF, FGF, and TGF‐β1 are emerging. Recently, NRPs have been described to play an integral role in vascular adhesion, regulating endocytosis, and EndMT in PDAC—factors that should be taken into account while developing novel therapies. Additionally, NRPs have been implicated in mediating EMT in other cancer types.^95^, ^96^ Recently, it was discovered that the EMT program is dispensable for metastasis, but induces chemoresistance in pancreatic cancer.^97^ Therefore, examining the role of NRP‐1 in EMT and associated chemoresistance in PDAC requires prompt attention. Targeting NRPs could provide both benefits and detriments due to their multifunctionality, as providing therapy for one symptom may give rise to adverse effects within or outside of the tumor environment. For example, treatment with a human anti‐NRP‐1 antibody in phase I clinical trial (Genentech, Inc., South San Francisco, California, USA.) was followed by transient platelet depletion, while combination therapy with an anti‐VEGF antibody (bevacizumab) resulted in proteinuria.^98^, ^99^ As such, a better understanding of their interactions with other ligands and downstream signaling pathways will help the development of effective therapies targeting PDAC. One of the earliest anticancer strategies involved the administration of soluble NRP‐1 variants that could function as a VEGF trap^100^ to decrease tumor angiogenesis and progression. Based on recent findings, it is likely that this strategy could block other growth factors as well, but this has not been examined. Teesalu et al^91^ observed that several cell‐penetrating peptides bind to NRP‐1 and possessed a C‐terminal consensus R/KXXR/K motif, preferentially with a C‐terminal arginine (R) or occasionally lysine (K), termed as the C‐end rule. These peptides appear to bind to the electronegative pocket of the b1 domain of NRP‐1. Interestingly, these peptides were internalized into the cells very rapidly. Accordingly, rather than neutralizing NRP, administration of internalized‐RGD peptide has displayed efficacy in binding to NRP‐1 to enhance penetration of a range of anticancer drugs.^93^, ^101^, ^102^ Additionally, peptide mimetic of the exon‐8 C‐terminal motif of VEGF_165_, called Tuftsin, which binds to the electronegative b1‐domain of NRP‐1 and competes with VEGF_165_for binding,^103^ can also be considered for future therapeutic strategies.

Ideally, further research into NRP‐2 will allow us to choose the best strategy in scenarios where NRP‐2, but not NRP‐1, is upregulated. Furthermore, the function of NRP‐1/NRP‐2 heterodimers should be investigated in PDAC as their similar but unique properties may confer different advantages to the tumor. Strategies to reduce NRP expression or to neutralize the receptors have been investigated, such as RNA interference (RNAi), specific monoclonal antibodies, and small peptides (Figure 2). Some of the benefits and shortcoming of these strategies have been described (Table 1). However, it may not be necessary to target NRPs in every pathological context. Perhaps, therapies focusing on ligands, such as SEMA3, could be considered as some isoforms contribute to the tumorigenicity of PDAC, while others function as inhibitors of the process as they compete with VEGF ligands for binding. Overall, novel therapies targeting NRPs in PDAC are worth pursuing because successful techniques could also be applied to other cancers where NRPs are highly expressed, such as non‐small‐lung carcinomas and breast cancers.^16^

**Table 1:** Various strategies for potential anti‐NRP cancer therapies against PDAC

Strategy	Advantages	Disadvantages	Mitigation approaches
RNA interference (siRNA, miRNA)	High target mRNA specificity Low toxicity, low immunogenicity Relatively inexpensive Already tested in human clinical trials^84^ Ease of delivery	Rapidly cleared by DNases Reduced transfection efficiencies in vivo^85^ Uptake by off‐target tissues^85^	Modified nucleotides (mimics) have increased stability^86^ Multiple deliveries to enhance therapeutic effects Target‐specific ultrasound‐mediated gene delivery
RNA interference (viral vector‐based shRNA)	High target mRNA specificity Prolonged transduction efficiency in vivo Already tested in human clinical trials^84^	Varying levels of toxicity and immunogenicity^87^ Expensive to make, time‐consuming Uptake by off‐target tissues Circulating virus‐neutralizing antibodies can reduce transduction efficiencies^87^ Risk of insertional mutagenesis	Conditionally replicating virus for tumor‐specific transduction^87^ Target‐specific ultrasound‐mediated viral delivery Nonintegrating lentiviral vectors to lessen the risk of random insertion
Monoclonal antibody	High receptor‐binding efficacy Already tested in human clinical trials^88^ Ease of delivery	Varying levels of toxicity and immunogenicity Expensive to make, time‐consuming^89^ Risk of binding to receptors on off‐target sites Monospecific monoclonal antibodies may be unable to provide the desired therapeutic effect^90^	Blockade of multiple factors and pathways using bispecific antibodies may result in improved therapeutic efficacy^90^ Can be coupled with drug‐loaded liposomes for enhanced therapeutic effect^91^
Drug‐loaded peptide	High specificity to tumor vessel endothelium and PDAC cells highly expressing NRP‐1^92^, ^93^ More effective than drug cotreated with peptide^94^	Possibly limited in clinical pancreatic cancer due to poor vascularity and high degree of CAFs^93^	Reduce abundance of CAFs by targeting EMT and/or EndMT
Hybrid lytic peptides	High receptor‐binding specificity and selectivity toward cancer cells^92^ Low manufacturing cost Low risk of immune response More effective penetration compared to antibody‐based therapies	Not yet tested in vivo and could still affect normal cells that express NRP‐1 Mechanism of peptide‐induced death not well understood	Further research into mechanism and therapeutic effects *in vivo is* warranted

The table summarizes the advantages, disadvantages, and some of the mitigation approaches for various potential anti‐NRP cancer therapies against pancreatic cancer. siRNA, silencing RNA; miRNA, microRNA; shRNA, short‐hairpin RNA; PDAC, pancreatic ductal adenocarcinoma; CAFs, cancer‐associated fibroblasts; EMT, epithelial‐mesenchymal transition; EndMT, endothelial‐mesenchymal transition.

## CONFLICT OF INTEREST

The authors declare no potential conflict of interests.
